# Restoring Study PRGF: a randomized clinical trial on plasma rich in growth factors for knee osteoarthritis

**DOI:** 10.1186/s13063-022-07049-3

**Published:** 2023-01-18

**Authors:** Luis Carlos Saiz, Juan Erviti, Leire Leache, Marta Gutiérrez-Valencia

**Affiliations:** 1 Unit of Innovation and Organization, Navarre Health Service, Pamplona, Spain; 2 IdiSNA, Navarra Institute for Health Research, Pamplona, Spain

**Keywords:** Plasma rich in growth factor, Platelet-rich plasma, Knee osteoarthritis, Restoring Invisible & Abandoned Trials (RIAT), Transparency, Research integrity

## Abstract

**Background:**

A randomized clinical trial assessing plasma rich in growth factors (PRGF) versus hyaluronic acid for knee osteoarthritis was published in 2012 (sponsor trial ID BTI-01-EC/07/ART). Evidence of misreporting was discovered following access to unpublished materials. In accordance with the principles of the Restoring Invisible and Abandoned Trials (RIAT) initiative, we sought to re-analyse Study PRGF based on the unpublished trial materials.

**Methods:**

Reanalysis was made possible primarily based on two unpublished study documents (original trial protocol and final report) obtained from the authors of the original publication. A call to action, calling on the authors to correct the original publication, was publicly issued. The involved ethics committee was repeatedly approached and extensive discussion with the authors ensued. After no agreement to correct the paper was reached, we embarked on this restoration. Reanalysis was focused on providing updated analyses for efficacy and safety.

**Results:**

The efficacy of PRGF was not statistically different from hyaluronic acid for any prespecified primary or secondary efficacy outcomes. For the primary endpoint, the percent of patients on PRGF compared to hyaluronic acid with a decrease >40% in WOMAC pain subscale score was 5.4% higher; 95% confidence interval (CI) −10.4% to 21.3%; *p* = 0.505. This differs from the original publication that reported a non-prespecified primary endpoint (decrease >50% in WOMAC pain subscale score) which was 14.1% higher; 95% CI 0.5 to 27.6%; *p*=0.044. Furthermore, in contrast to the article statement that all the adverse events disappeared in 48 h, at least two patients in the hyaluronic arm and five patients in the PRGF arm reported persistent adverse events. Inadequate disclosure of conflicts of interest in the original publication was also noted.

**Conclusions:**

This reanalysis of Study PRGF found no clinically or statistically significant benefit from PRGF compared to hyaluronic acid. The restoration of Study PRGF shows the urgency of important changes to trial reporting and oversight practices. In the future, timely access to all clinical trial documents is needed to minimize the risk of reporting bias. Similarly, ethics committees should be ready to intervene whenever a case of potential misconduct arises.

**Trial registration:**

This is a RIAT project, whose original trial was approved and registered on 19 December 2007 by the Ethics Committee of the Basque Country, Spain, as BTI-01-EC/07/ART.

**Supplementary Information:**

The online version contains supplementary material available at 10.1186/s13063-022-07049-3.

## Background

There is substantial concern within the scientific community about the profusion of incompletely reported and misreported studies. Projects such as the Restoring Invisible & Abandoned Trials (RIAT) initiative (https://restoringtrials.org), which was launched in 2013 [1], are aimed at providing a more accurate picture of the evidence with medicines and, consequently, a better care of patients by clinicians. The basic procedures consist of getting access to the underlying data of a trial, comparing the results with those published in journals, contacting the original authors to discuss discrepancies and correct the publication if errors are demonstrated, to promote a more accurate dissemination of the effects of an intervention [2]. With assistance from the RIAT Support Center (currently housed at the University of Maryland School of Pharmacy), several research teams have been able to conduct trial restorations [3]. The reanalysis of paroxetine Study 329 is probably the most recognized RIAT project thus far [4]. Nevertheless, globally considered just a small number of reanalyses of randomized clinical trials have been conducted and published by entirely independent researchers [5].

In this article, we present the results of our RIAT reanalysis of a clinical trial on plasma rich in growth factors (PRGF) known as BTI-01-EC/07/ART, hereafter referred to as Study PRGF. The original study was sponsored by BTI Biotechnology Institute (BTI). We acknowledge the work of the original investigators. This double-blind, randomized controlled trial was designed to assess the efficacy and safety of PRGF compared with hyaluronic acid for adults suffering from symptomatic knee osteoarthritis. It was reported in *Arthroscopy: The Journal of Arthroscopic and Related Surgery* in 2012 by Mikel Sánchez et al. [6]. Early in 2019, our publicly funded research team, hereafter referred to as the RIAT researchers, was commissioned to evaluate the evidence on the efficacy and safety of platelet-rich plasma (PRP) and analogues in traumatology. As a result of the literature review, the high-impact Study PRGF was retrieved and closely analysed. The publication by Sánchez and colleagues claimed superiority for PRGF over hyaluronic acid in alleviating symptoms of mild to moderate knee osteoarthritis with a similar safety profile. However, this conclusion was at odds with the full dossier of the clinical trial. This was concerning, in particular because the article had a large influence on practice by supporting the use of PRP and its derivatives at a time when these products were not widely known. Based on that, our main objective was to re-analyse Study PRGF according to the full available information for the trial.

Chronologically, on 8 January 2019, the RIAT researchers asked the study’s corresponding author for the related protocol, final report and Ethics Committee resolutions, which were granted on 14 January 2019. Once the dossier was reviewed, the RIAT researchers wrote again on 21 January 2019 to explain in detail every detected concern, asking for clarification. From that time on, two phone calls and twelve emails were exchanged. In accordance with RIAT procedures, the RIAT researchers made public on 27 March 2019 the need to correct the article and simultaneously informed the contact author about this ‘call to action’ [7]. The study authors responded by email on 3 June 2019, refusing any responsibility and stating that the Ethics Committee endorsed them. The ethics committee provided a letter on 20 June 2019. The RIAT researchers tried repeatedly to get information directly from the Ethics Committee but failed. Considering that this alleged support did not solve the major misreporting identified in the article, the RIAT researchers reiterated the need of a more substantial correction; the authors rejected this proposal on 26 June 2019.

Study PRGF was a multicentre, 24-week, double-blind, randomized controlled trial [6]. According to the protocol, BTI’s declared objective was to evaluate the short and medium-term efficacy and safety of PRGF in the symptomatic treatment of knee osteoarthritis. Secondary aims were to investigate biological markers in peripheral fluids that may reflect time phases of disease progression. The ultimate goal was to assess disease evolution and monitor treatment through objective biological outcomes. The hypothesis was that PRGF would improve pain symptoms compared with HA in patients affected by knee degeneration. Study recruitment of patients took place between January 2008 and November 2009, and the study was completed on September 2010 [6].

## Methods

We reassessed the data of Study PRGF in accordance with the RIAT recommendations. To this purpose and apart from the involved published article, we used as principal sources the following documents: (1) full original protocol, (2) ethics committee approval resolution, (3) final study report (summary version). All of them were accessed on request from the contact author of the original publication. Other related documents (Statistical Plan Analysis, study database, set of documents reviewed by the Ethics Committee and alleged final report dated November 2011) were also requested from the study authors with no response from their side.

According to RIAT recommended procedures [1], our methods are those previously set out in the January 2008 protocol for Study PRGF. When the methods used and published by Sánchez and colleagues differed from the protocol, we followed the original protocol. No official amendments to the protocol have ever been proved. In cases when the protocol was not precise enough, we decided by consensus standard methods to present the data in an appropriate way and inform explicitly on our decision.

### Participants

A total of 187 participants were recruited at three clinical sites in the Basque Country (Spain). Potential candidates were informed in a first visit about the aims and methods of the clinical trial. Those patients showing interest in the trial were asked to sign an informed consent document. Basal blood and radiological tests were carried out whenever needed. All inclusion and exclusion criteria (Table 1) had to be met before entering the study in a subsequent ‘diagnostic visit’. Once included, each participant received a booklet to be filled in at home, including the Western Ontario and McMaster Universities Osteoarthritis Index (WOMAC) questionnaire. As a result of this process, 176 participants were eventually randomized to the pre-planned interventions.

**Table 1:** Inclusion and exclusion study criteria according to the protocol body text

**Inclusion criteria**	
• Male and female adults aged 40–72 years	
• Radiological diagnosis of tibiofemoral knee osteoarthritis	
• Joint pain >35 mm, according to Visual Analogue Scale (range 0–100 mm)	
• Radiological severity <4, according to the Ahlbäck scale (range 1–5)	
• Body Mass Index between 20 and 30 ^**a**^	
• Availability to be examined along the follow-up period	
**Exclusion criteria**	
• Bilateral knee osteoarthritis needing infiltration in both knees	
• Body Mass Index ≥30 ^**b**^	
• Diagnosis of polyarticular disease	
• Severe mechanical deformity (bidiaphyseal varus 4° and valgus 16°)	
• Previous arthroscopy within last year	
• Hyaluronic acid infiltration within last 6 months	
• Diagnosis of systemic autoimmune rheumatoid disease (connective tissue diseases and systemic necrotizing vasculitis)	
• Uncontrolled diabetes mellitus (glycosylated haemoglobin >7%)	
• Blood disorders (thrombopathy, thrombocytopenia, anaemia with haemoglobin <9)	
• Ongoing immunosuppressive and/or dicumarinic therapies	
• Use of corticosteroids within last 3 months before inclusion in study	
• Use of non-steroidal anti-inflammatory drugs within the last 15 days before inclusion in the study	

^a^According to the summary of the protocol, final report and published article, body mass index between 20 and 32

^b^According to the summary of the protocol, body mass index ≥32. According to the final report and published article, body mass index ≥33

### Interventions

Each included participant was randomized to receive infiltrations of the affected knee (3 injections on a weekly basis) with either PRGF-Endoret® (BTI) or hyaluronic acid (Euflexxa®, Copenhagen, Denmark). PRGF-Endoret® was prepared from autologous peripheral blood at each treatment visit by following subsequent steps of blood extraction, centrifugation and plasma fractioning. Plasma rich in platelets without leukocytes was then pipetted and activated with calcium chloride before infiltrated. Compliance was not considered a useful outcome to be estimated due to the fact that both interventions were administered by the researchers. Acetaminophen intake, the only permitted medication for pain relief throughout the trial period, was registered during the trial.

### Sample size

Sample size calculation was initially stated in the protocol based on the original main outcome, the percentage of patients with a decrease >40% in the score in the WOMAC pain subscale at the final visit with respect to baseline (which is similar to a pain improvement >40%). Null hypothesis was set as no difference in that outcome between both interventions. A statistical power of 80% and *α*=0.05 were established, comparing two proportions by using the bilateral *X*^2^ test for independent samplings. Assuming that the proportion of participants reaching the main outcome was expected at 25% in the experimental group versus 10% in the control group, a total of 102 participants were estimated per arm. An anticipated dropout rate of approximately 5% was then considered, setting the final number of participants to 222 (111 per group). However, in the published article [6], the key parameter for the primary outcome measure was changed (decrease >50% instead of >40%) without amending the sample size calculation. In addition, study power (90%) and assumed proportion of patients achieving the main outcome (30% in the experimental group and 12% in the control group), was also amended without any changes to the sample size calculation. The final report did not inform on the potential reasons behind the gap between the calculated sample size and the total number of enrolled patients (187 participants), which may indicate that the trial was in practice underpowered.

### Randomization and blinding

A stratified randomization in blocks of four was performed by using a specific software developed by GlaxoSmithKline (C4 Study Design Pack). Each centre was considered as one stratum. Randomized numbers were generated and assigned to participants, which were subsequently allocated to one study group, PRGF or hyaluronic acid. Sealed envelopes were used to conceal treatment allocation until the moment before applying the treatment.

Both participants and independent outcome assessors remained blinded to treatment assignment. Clinical researchers were not blinded due to relevant differences between both interventions in terms of appearance and viscosity. As for participants, peripheral blood was drawn from all patients regardless their assigned treatment and the infiltration area was hidden while the injection was given. Outcome assessors were not involved in infiltrating included participants.

### Outcomes

According to the original protocol, participants were expected to be assessed 1, 2, and 6 months after the last treatment infiltration for the following outcomes:

#### Primary efficacy outcome

The prespecified primary efficacy outcome was a clinically significant improvement in pain, measured as the percentage of patients with a decrease >40% in the score in the WOMAC pain subscale at the final visit with respect to baseline. This outcome was not presented in the published article but only in the unpublished final report.

#### Secondary efficacy outcomes

The prespecified secondary efficacy outcomes were as follows:

- Clinically significant improvement in pain, measured as the percentage of patients with a pain decrease >40% in the score in the Visual Analogue Scale (VAS) and Lequesne index at the final visit with respect to baseline. These outcomes were not presented in the published article but they were in the unpublished final report.
- Clinically significant improvement in physical function, measured as the percentage of patients with a decrease ≥30% in the score in the Lequesne index and WOMAC scale at the final visit with respect to baseline. These outcomes were not presented either in the published article or in the final report.
- Quality of life (questionnaire SF-12). This outcome was not presented either in the published article or in the unpublished final report.
- Degree of joint mobility, measured by using a goniometer. This outcome was not presented in the published article but it was in the unpublished final report.
- Use of acetaminophen. This outcome was presented in the published article and also in the unpublished final report.
- Investigational biologic markers in plasma and synovial liquid; inflammatory markers as C-reactive protein; anabolic and angiogenic growth factors; synovial function markers as hyaluronic acid and YKL-40; indicators of cartilage anabolic/catabolic imbalance. These outcomes were not presented either in the published article or in the unpublished final report.

Leaving aside the aforementioned pre-planned outcomes, the study authors included in the published article several primary and secondary outcomes not prespecified in the original protocol (Table 2). We could not find any document that provided any scientific rationale for these post hoc changes, and the outcomes are therefore not reported in detail in this paper.

**Table 2:** Prespecified and published primary and secondary outcomes

	Was it included in the original protocol?	Was it included in the final report?	Was it included in the published article?	Were any statistically significant differences found?
**Primary outcome**				
% of patients with a pain decrease **>40%** in the score in the WOMAC pain subscale	Yes	Yes	No	No
% of patients with a pain decrease **>50%** in the score in the WOMAC pain subscale	No	No	Yes	Yes
**Secondary outcome**				
% of patients with a pain decrease >40% in the score in the VAS and Lequesne index	Yes	Yes	No	No
% of patients with a function increase ≥30% in the score in Lequesne and WOMAC	Yes	No	No	---
Quality of life (SF-12 questionnaire)	Yes	No	No	---
Degree of joint mobility (goniometer)	Yes	Yes	No	No
Use of acetaminophen	Yes	Yes	Yes	No
Investigational and inflammatory markers. Indicators of cartilage metabolic imbalance	Yes	No	No	---
OMERACT-OARSI responders	No	No	Yes	No
20% decrease in WOMAC pain score	No	No	Yes	No
% change normalized WOMAC pain score	No	No	Yes	No
% change normalized WOMAC stiffness score	No	No	Yes	No
% change normalized WOMAC function score	No	No	Yes	No
% change normalized WOMAC total score	No	No	Yes	No
% change Lequesne index	No	No	Yes	No

*VAS* Visual Analogue Scale, *WOMAC* Western Ontario and McMaster Universities Osteoarthritis Index, *OMERACT-OARSI* Outcome Measures in Rheumatology Clinical Trials-Osteoarthritis Research Society and Health Assessment Questionnaire

#### Safety outcomes

A record of complications and/or adverse events with imputation scale was performed. In accordance with the protocol, any sort of circumstances considered by researchers as an adverse event was to be recorded in detail in the booklet, including surgical or postsurgical complications. Non-severe adverse events or those not considered as related to interventions were expected to be listed within the annual report and/or final report.

### Statistical analysis

With regard to the statistical analysis, RIAT investigators adhere to the original protocol procedures linked only to primary and secondary prespecified outcomes. A database was created to register and extract data from every endpoint described in the study protocol, including a statistical analysis when needed [8] (see Additional file 1). Thus, any other exploratory analysis has been discarded from our reanalysis unless a compelling justification is explicitly offered. A descriptive analysis of the sample was performed, providing with demographic and clinical data. Quantitative outcomes, such as body mass index (BMI) and age, were estimated by reporting the mean, standard deviation and range. Regarding the qualitative ones, such as sex, marital status, education level, physical activity, medical history, medications and osteoarthritis radiologic severity, a frequency analysis was carried out. Basal comparability for both intervention groups was tested. The efficacy outcomes were assessed by using either a *X*^2^ test or a Student’s *t* test, depending on their qualitative or quantitative status. Beyond the scope of our re-analysis, focused on clinical efficacy and safety outcomes, several secondary variables were assessed by an analysis of covariance including intervention, centre and radiological severity as factors and BMI, age and acetaminophen consumption as covariates.

Efficacy analyses were prespecified to be conducted using a *per-protocol* (PP) approach, which was followed in the unpublished final report. The protocol also mentioned an *intention-to-treat* (ITT) approach to be used in dropout analysis. This latter alternative was chosen by the study authors to report the outcome results in the published article. In order to keep a conservative strategy, RIAT investigators decided to provide both analyses where possible. Regarding safety outcomes, all adverse events were individually described and a frequency analysis by intervention arm was compared by performing a *X*^2^ test. A blood test was planned one month after the last treatment infiltration. All statistical analyses were carried out by the sponsor.

Missing values were jointly dealt by the sponsor, lead researcher and statistician to decide on the last meaningful data (probably a *last observation carried forward* method but not confirmed) for any individual case.

The final unpublished report was used as the main source of data for all outcomes except the non-preplanned primary outcome and use of acetaminophen, as data for these variables were only available in the article publication.

## Results

Table 3 presents the baseline characteristics of the intervention groups, including those already showed in the original article [6] (see Table 2, page 1074) and other variables (marital status, education level, Lequesne/VAS pain subscales and degree of joint mobility) only communicated in the final report (pages 13–14). No imbalance was detected for any characteristic taken into consideration.

**Table 3:** Basal characteristics according to the journal article and unpublished final report

	PRGF	HA	***p*** value
**No. of participants**	89	87	
**Age (y)**, mean (SD)	60.5 (7.9)	59.0 (8.2)	0.198
**Sex (% female patients)**, *n* (%)	46 (52)	45 (52)	0.996
**Body mass index (kg/m**^**2**^**)**, mean (SD)	27.9 (2.9)	28.2 (2.7)	0.590
**Dose of acetaminophen (mg/d)**, median (range) ^**a**^	2.6 (7.1)	1.7 (5.6)	0.631
**Normalized WOMAC score Pain subscale**, mean (SD)	40.4 (16.3)	38.4 (15.6)	0.417
**Normalized WOMAC score Stiffness subscale**, mean (SD)	41.8 (17.3)	38.5 (18.3)	0.233
**Normalized WOMAC score Physical function subscale**, mean (SD)	39.6 (16.3)	38.8 (17.4)	0.755
**Normalized WOMAC score Global**, mean (SD)	121.8 (44.4)	115.7 (45.1)	0.378
**Lequesne index**, mean (SD)	9.5 (3.0)	9.1 (3.2)	0.408
**Lequesne index—pain**, mean (SD)	4.5 (1.5)	4.1 (1.6)	0.083
**Visual Analogue Scale—pain**, mean (SD)	5.5 (1.5)	5.7 (1.5)	0.281
**Degree of joint mobility**, mean (SD)	126.5 (10.2)	125.3 (17.0)	0.576
**Primary arthritis**, *n* (%)	73 (82)	68 (78)	0.521
**Ahlbäck grade I**, *n* (%) ^**b**^	45 (51)	42 (48)	0.973
**Ahlbäck grade II**, *n* (%) ^**c**^	32 (36)	32 (37)	
**Ahlbäck grade III**, *n* (%)	12 (13)	11 (13)	
**Marital status—single**, *n* (%)	7 (8)	4 (5)	0.676
**Marital status—married**, *n* (%) ^**d**^	69 (78)	66 (76)	
**Marital status—widow/widower**, *n* (%)	6 (7)	9 (10)	
**Marital status—divorced**, *n* (%)	7 (8)	8 (9)	
**Education level—Group 1**, *n* (%)	5 (6)	1 (1)	0.242
**Education level—Group 2**, *n* (%)	42 (47)	44 (51)	
**Education level—Group 3**, *n* (%)	27 (30)	25 (29)	
**Education level—Group 4**, *n* (%)	12 (13)	9 (10)	
**Education level—Group 5**, *n* (%)	3 (3)	8 (9)	

*HA* hyaluronic acid, *PRGF* plasma rich in growth factors, *SD* standard deviation, *y* years, *WOMAC* Western Ontario and McMaster Universities Osteoarthritis Index. All data from the unpublished final report except dose of acetaminophen and normalized WOMAC score stiffness and physical function subscales. Figures rounded to one decimal place. *p* values provided by the study authors in the journal article and unpublished final report

^a^According to the published article (footnote in Table 2), dose of acetaminophen does not fit with the units (mg/d) and the figures shown in that table

^b^HA (%) presented in the final report has been corrected, 48 instead of 49

^c^HA (%) presented in the final report has been corrected, 37 instead of 38

^d^PRGF (%) presented in the final report has been corrected, 78 instead of 77

A total of 176 participants underwent randomization (PRGF=89, HA=87). Reasons for exclusion before randomization included BMI out of range, high radiological severity and knee deformity (*genu varus*). After randomization, subjects were excluded because of non-permitted non-steroidal anti-inflammatory drugs (NSAID) consumption, steroid infiltrations or other surgical procedures not allowed by protocol. A total of 10 patients from the PRGF arm (11.2%) and 13 from the HA arm (14.9%) were excluded/withdrawn after being randomized. There have been registered three different reasons for exclusion: NSAID consumption (4 in the PRGF arm; 3 in the HA arm), corticosteroid infiltration (1 in the PRGF arm; 5 in the HA arm), and surgery procedures (2 in the PRGF arm; 1 in the HA arm). Also, some participants (2 in the PRGF arm; 4 in the HA arm) decided to withdraw their consent mainly due to perceived uselessness. Generally speaking, a visual inspection does not reveal relevant imbalances beyond a more frequent use of corticoid infiltrations in the hyaluronic acid arm.

### Efficacy

A per-protocol efficacy analysis was defined in the protocol (page 41), which was coherent with the final unpublished report on this issue. However, the corresponding publication reported an intention-to-treat analysis instead of the pre-planned per-protocol analysis. Table 4 shows the per-protocol and intention-to-treat analyses for all pre-planned primary and secondary outcomes with available results, plus the non-preplanned primary outcome (percentage of patients with a decrease >50% in the score in the WOMAC pain subscale). There was a crucial discrepancy affecting the primary outcome between our analysis, which remained faithful to the protocol and final unpublished report documents, and that published by the study authors. As Table 4 displays, no statistically significant differences between both arms were identified for any primary or secondary pre-planned outcome when the final unpublished report is considered. When a decrease >50% in the WOMAC pain subscale was selected as the main outcome in a post hoc approach, inconclusive evidence from PP and ITT analyses could be drawn. In addition, the confidence interval shown by the only statistically significant result 14.1% (0.5–27.6) is wide enough as to likely include non-clinically significant differences. The protocol listed several pre-planned secondary outcomes (% patients with a function increase ≥30% in the score in Lequesne and WOMAC, quality of life, investigational/inflammatory markers and indicators of cartilage metabolic imbalance) which sadly were not available from any source.

**Table 4:** Primary and secondary outcomes in per-protocol and intention to treat population ^a^

**Primary outcome**		**PRGF**	**HA**	**Proportion mean difference (95% CI)**	***p*** **value**
		**n/N (%)**			
% of patients with a decrease **>50%** in the score in the WOMAC pain subscale (non-preplanned)	PP analysis	34/78 (43.6%)	21/74 (28.4%)	15.2% (0.2 to 30.3)	0.051
	**ITT analysis**	**34/89 (38.2%)**	**21/87 (24.1%)**	**14.1% (0.5** to **27.6)**	**0.044**
% of patients with a decrease **>40%** in the score in the WOMAC pain subscale ^**b**^ (preplanned)	**PP analysis** ^**c**^	**39/78 (50.0%)**	**33/74 (44.6%)**	**5.4% (−10.4** to **21.3)**	**0.505**
	ITT analysis	39/89 (43.8%)	33/87 (37.9%)	5.9% (−8.6 to 20.4)	0.427
**Secondary outcomes**		**n/N (%)**		**Proportion mean difference (95% CI)**	***p*** **value**
% of patients with a decrease >40% in the score in the VAS ^**b**^	PP analysis ^**c**^	36/79 (50.0%)	36/74 (44.6%)	−3.1% (−18.9 to 12.7)	0.206
	ITT analysis	36/89 (40.4%)	36/87 (41.4%)	−0.9 (−15.5 to 13.6)	0.900
% of patients with a decrease >40% in the score in the Lequesne index ^**b**^	PP analysis ^**c**^	53/77 (68.8%)	43/73 (58.9%)	9.9 (−5.4 to 25.2)	0.206
	ITT analysis	53/89 (59.6%)	43/87 (49.4%)	10.1 (−4.5 to 24.8)	0.177
**Secondary outcomes**		**Mean (95% CI)**		**Mean difference (95% CI)**	***p*** **value**
Degrees of knee flexion	PP analysis ^**c**^	126.6 (123.2–129.9)	124.8 (121.4–128.1)	1.8 (−2.9 to 6.5)	0.447
Visit 1 (1 month)					
Degrees of knee flexion		128.1 (125.4–130.9)	128.4 (125.6–131.2)	−0.3 (−4.2 to 3.6)	0.883
Visit 2 (2 months)					
Degrees of knee flexion		128.4 (125.6–131.1)	128.8 (126.0–131.6)	−0.5 (−4.4 to 3.4)	0.809
Visit 3 (6 months)					
Use of acetaminophen^**a,d**^ (g/day)	ITT analysis	0.1 (2.0)	0.1 (2.3)	---	0.853

*CI* confidence interval, *g* grams, *HA* hyaluronic acid, *ITT* intention-to-treat, *PP* per-protocol, *PRGF* plasma rich in growth factors, *VAS* Visual Analogue Scale, *WOMAC* Western Ontario and McMaster Universities Osteoarthritis Index

^a^A per-protocol analysis [>50% in the WOMAC pain subscale] and three intention-to-treat (ITT) analyses [>40% in the WOMAC pain subscale; >40% in the VAS; and >40% in the Lequesne index] have been estimated and presented by our team to let readers know about potential differences in dichotomic primary and secondary outcomes when an alternative strategy of analysis is performed. This information can help to evaluate the discrepancy between the analysis strategy planned in the protocol (a per-protocol analysis) and the one followed in the original publication (an ITT analysis)

^b^Outcome defined in the study protocol as >40% but results in the final report informed as ≥40%

^c^Not adjusted results with standard errors for the continuous variables and number of events with *p* values for dichotomous outcomes are provided in the final report. Based on that, standard deviations and subsequent confidence intervals have been calculated and presented for a per-protocol analysis. An ITT analysis was also performed for the continuous variable (PRGF, *n*=89; hyaluronic acid, *n*=87), which results are not shown to avoid redundancy as they were essentially similar to the PP analysis

Results for several pre-planned secondary outcomes [% patients with a function increase ≥30% in the score in Lequesne and WOMAC, quality of life, investigational / inflammatory markers and indicators of cartilage metabolic imbalance] are not available

^d^Use of acetaminophen (g/day) expressed as median and range, only for the intention-to-treat analysis. Additional data from the final report were not considered for publication because of information incompleteness, which prevents us from providing reliable data

### Harms

Table 5 presents the number of adverse events found in this study summarized by type of analysis, Ahlbäck grade and relation to treatment in accordance with researchers’ assessment. Around one in three participants suffered an adverse event, with no differences between both treatment groups regardless the analysis performed (per-protocol or intention-to-treat). Serious or unexpected adverse events were rare and equally distributed between the PRGF and HA groups.

**Table 5:** Adverse events registered in the clinical trial

Safety outcomes	PRGF		HA	Proportion mean difference (95% CI)	***p*** value
	n/N (%)				
**Total AE**^**a**^	**PP analysis**	26/79 (32.9%)	24/74 (32.4%)	0.5% (−14.4 to 15.3)	0.950
	**ITT analysis**	26/89 (29.2%)	24/87 (27.6%)	1.6% (−11.7 to 14.9)	0.811
**Serious or unexpected AE**^**b**^	1/26 (3.8%)		1/24 (4.2%)	---	---
**Total persistent AE** ^**c**^	5/26 (19.2%)		2/24 (8.3%)	---	---
**Total AE by Ahlbäck grade:**				---	---
Grade 1	17/26 (65.4%)		12/24 (50%)		
Grade 2	4/26 (15.4%)		11/24 (45.8%)		
Grade 3	4/26 (15.4%)		1/24 (4.2%)		
Grade 4	1/26 (3.8%)		0/24 (0.0%)		
**Total AE by relation to treatment:**				---	---
Unrelated	25/26 (96.2%)		22/24 (91.6%)		
Possible	0/26 (0.0%)		1/24 (4.2%)		
Highly likely	1/26 (3.8%)		1/24 (4.2%)		

*AE* adverse event, *HA* hyaluronic acid, *ITT* intention-to-treat, *PP* per-protocol, *PRGF* plasma rich in growth factors

^a^Proportion mean differences for the outcome ‘Total AE’ have been calculated and presented using the raw data provided by the final report

^b^There were registered one participant with a knee trauma event during the study in the PRGF group and one participant with a non-specific trauma event in the HA group

^c^There were registered four participants with knee pain and one participant with shoulder pain in the PRGF group. One participant with knee pain and one participant with knee and hip pain were registered in the HA group

Regarding causality, local researchers judged most of recorded adverse events (>90%) unrelated to the interventions. It should be recognized that assessing causality is always a challenging task. Nonetheless, further clarification on the followed criteria to determine a potential relation is lacking, particularly when this judgement can be seen as highly subjective. For example, there were eleven participants in the study who suffered from knee pain as an adverse event [eight participants in the PRGF arm (four of them persistent) and three in the hyaluronic arm (one of them persistent)]. Beyond the higher numerical prevalence of this adverse event (AE) in the PRGF arm, there remain some concerns on how a relation to the intervention was fully discarded for all but one case.

Finally, persistent adverse events were numerically more common in the PRGF arm (5 out of 26 AEs) than in the HA acid arm (2 out of 24 AE). Even if these figures cannot imply a real difference between both arms, they are not in line with the authors’ statement when declared in the publication that all adverse events disappeared in 48 hours.

## Discussion

### Main findings and contrast with original published article

Our RIAT appraisal of Study PRGF underlined that no substantial differences in pain relief and other efficacy estimates were proved when adult participants with knee osteoarthritis were treated with plasma rich in growth factors or hyaluronic acid. As far as safety is concerned, there is no sound evidence about a different profile between both interventions, while some data on type and persistent adverse events make advisable further research. This analysis is well aligned with the final report authored by Sánchez and colleagues but it is not the case concerning the published article in *Arthroscopy* [6].

We assessed and reported the Study PRGF in accordance with the original protocol, which had no amendments reported by the study authors or the corresponding ethics committee. The re-assessment of the dossier has shown a marked discrepancy in the main efficacy outcome based on the fact that unlike study authors, in the analysis the RIAT investigators stuck to the protocol and original final report. A specific section in the protocol devoted to ethical considerations (page 35) clearly stated that ‘no changes or deviations of the protocol will be permitted without evidence of approval’. However, the authors reported a non-approved main efficacy outcome, which should be seen as a protocol major change, reaching statistically significant differences in favour of the investigational product. It has been previously described that changes to endpoints can compromise the scientific integrity of a trial, leading to misguided research and suboptimal patient care [9].

Our re-analysis has shown in detail how an inappropriate shift in a primary outcome can dramatically influence final results. In this sense, it should be noted that the original statistical analysis was performed by the own trial sponsor, which stresses the need to rely on fully independent bodies for this crucial task.

Other issues such as secondary outcomes reported but not pre-specified and vice versa and the unexpected change in the analysis plan from a per-protocol to an intention-to-treat strategy were also identified. Theoretically, such a switch might have come from the journal review process or a late realization from the original team that intention-to-treat was most appropriate. However, the change should have been explained and justified in the original work, as should the switch of the primary outcome from 40% to 50% reduction. Interestingly, as Table 4 indicates, not only a shift in the original main outcome resulted in statistically significant differences between both interventions but also was needed a change from the preplanned per-protocol strategy to an intention-to-treat analysis. As it has been shown previously, up to thirteen secondary outcomes were either preplanned in the protocol or included in the published report. Despite that, no mention to the need for multiple testing correction has been found. Nonetheless, there were no statistically significant findings to be emphasized for any secondary outcome, so in practical terms corrections were not essential for this analysis.

With regard to harms caused by the interventions, inconsistencies between text and figures appeared on persistent symptoms. Finally, the study authors failed to report their conflicts of interest in the authorship and publication of this article. In particular, one of them (EA) was the Scientific Director of the BTI Biotechnology Institute, manufacturer of PRGF-Endoret®, and others (JJA, SP, GO) were employees of the company [6].

### Agreements and disagreements with other studies

Beyond the clinical trial carried out by Sánchez et al. [6], it is not uncommon to find other studies [10–13] and even systematic reviews [14] claiming that PRPs and PRGFs are safe and effective. However, an overview on platelet-rich plasma injections for knee osteoarthritis published in 2019 by the National Institute for Health and Care Excellence (NICE) declared that, despite no major safety concerns have risen, ‘evidence on efficacy is limited in quality’, ‘long-term clinical effectiveness is unknown’ and ‘outcome measures of self-reported pain relief and knee function are subjective and may be confounded by various factors’ [15].

When studies are examined individually, there are important and repeated weaknesses: no randomization [12, 13], no placebo group [6, 10–13, 16], low patients number [10–13, 16, 17], short follow-up period [12], lack of blinding [11–13, 16] or conflicts of interest [6, 10, 11], among others. Even the selected comparator in many of these trials, hyaluronic acid, might not have been a wise decision. NICE is not recommending the use of intra-articular hyaluronic acid injections since 2014, given its relatively short intra-articular persistence estimated from just a few hours to days [18]. Bearing in mind all these aspects, we can gather that uncertainty is still the prevailing situation. Our research results match well with this statement, providing evidence of a supposedly poorly driven clinical trial within those a priori more prominent studies in its field.

### RIAT procedures

Compared to previous exercises, basically represented by the Study 329 [4], this RIAT process has probably entailed less complexity in its analyses but a similar demanding effort and perseverance. The dialogue with the study authors extended over 6 months and 16 total email messages. At first, gaining access to the protocol, final report and ethics committee approval was not an issue but the same could not be said of other relevant data. In particular, the specific Statistical Analysis Plan, the full set of documents reviewed by the ethics committee and the individual patient data, as well as a claimed but never facilitated report dated November 2011, were requested and unfortunately not provided by the study authors. On the other hand, we can confirm that *Trials* has had the opportunity to review all available information. Readers interested in verifying our analysis are strongly encouraged to contact the corresponding author of the original publication to obtain the original documents. Additionally, we share the Study 329 RIAT team’s view that individual patient level data are crucial when assessing adverse events [4]. To a certain extent, this lack of information could make our analysis less informative than intended. Nonetheless, it strongly underlines the necessity of a much wider transparency policy when a clinical trial is published.

Following the RIAT principles, the study authors were offered from the beginning the possibility to correct themselves all detected inaccuracies. A call to action was publicly issued and sent to the study authors [7]. Once clearly established their refusal to make any amendment, the journal where the trial was originally published was contacted and appropriately informed. As we have documented in a past publication [19], journals are often reluctant to correct or retract their articles even when serious flaws have been proved. In this particular case, the journal finally discarded any kind of retraction, amendment or re-publication process and invited our team to publish a letter summarizing our main concerns [20], which was subsequently replied by the study authors [21] and accompanied by an editors’ note.

The Osteoarthritis Research Society International (OARSI) assessed in 2000 the optimal cut-offs to be applied for the OARSI responder criteria regarding several settings, including knee intra-articular specific drugs for osteoarthritis [22]. The prioritized proposal (proposition A) estimated a 40% relative change as high improvement in pain for these drugs. Four years later, the performance of the OARSI 2000 sets of criteria was evaluated and a simpler set of responder criteria across all settings was adopted for osteoarthritis, which included a high improvement in pain or in function ≥50% and absolute change ≥20 points [23]. Given this context, the authors reasoned in their letter in favour of using the OARSI 2004 Guideline [23]. However, this document was published 4 years before the study protocol was completed and its recommendations did not restrict the condition of ‘responder’ to pain improvement by at least 50%, but they also added the need to cause a change in the WOMAC pain subscale of at least 20 points in absolute terms [23]. Thus, there was no time justification for adopting the OARSI Guideline recommendation in 2010/2011, once the study was finished, nor was the OARSI recommendation taken up in its entirety by the authors. Additionally, it is not clear why the impact of the supposedly harder goal (50% vs 40%) affected in a much greater extent to the control group than to the experimental one, as Table 4 shows.

And fourth, we would be pleased if our experience led to a deep reflection on the future role of ethics committees, reinforcing their commitment with the best practices in research. To fulfil this important mission over the entire life of a clinical trial, these committees would need adequate and well-trained resources, as well as the ability to take action when a case of malpractice is detected.

### Strengths and limitations

Study PRGF was a randomized controlled trial with a modest sample size, although larger than most studies in its field [10–13, 16, 17]. The trial maximum follow-up was only 6 months, not being useful to provide information on long-term outcomes, of great importance in chronic conditions such as osteoarthritis. According to the Ahlbäck score, 87% of included participants had a radiological severity <3, which is focused on those patients with less impairment, limiting the generalizability of the results. Additionally, it must be taken into account that, in normal practice, PRPs and its derivatives are mostly used in high-severity patients as a last resort, a setting far from that investigated in the present study.

Beyond the specific characteristics of the trial, evidence of several protocol violations has been proved over the RIAT process, clearly undermining the results showed by the original journal publication. On the other side, the RIAT development has been feasible due to the access to two major sources of data (protocol and final report) but it must be recognized that our re-analysis was also limited mainly related to the lack of individual participant data. This information was formally requested with no response received from the authors, which has prevented us from presenting results in more detail but also from discarding other potential problems such as data fabrication. Nonetheless, having in mind these limitations, we remain fully confident on the support that the existent data confer to our results.

## Conclusions

In contrast to the original published article by Sánchez and colleagues, our analysis of Study PRGF (originally coded as BTI-01-EC/07/ART) found no statistically significant differences between plasma rich in growth factors and hyaluronic acid in adults with knee osteoarthritis on any of the prespecified outcomes, included the primary efficacy variable. Serious or unexpected adverse events were rare and equally distributed between both interventions. Notwithstanding, further research should focus on finding out the real rate of persistent harms and the particular profile of adverse events linked to each treatment. Health professionals, especially in this case orthopaedic surgeons, but also researchers and decision-makers should be aware of the potential weaknesses in a priori high-quality research. It is highly convenient that all systematic reviews including the present clinical trial update their results in the light of this new evidence. In the same way, any other clinical trial or review based to some extent on the findings of this study is also called to re-assess its conclusions. Also, sharing the imperative demand by many other research teams, health authorities are strongly encouraged to make compulsory free access to protocols and raw data supporting subsequent publications.

As Le Noury and colleagues rightly stated in their pioneering report [4], ‘additional outcome variables outside those in the protocol cannot be legitimately declared once the study is underway except as exploratory variables [we would add that much less when the study has ended]’. ‘The a priori protocol and blinding are the bedrock of a randomised controlled trial [we would add that unfortunately, sometimes the bedrock turns into a dangerous sand foundation]’. ‘The primary mandate of the RIAT enterprise is to reaffirm essential practices in randomised controlled trials [we would add that we hope to have contributed to this goal at least to a small extent]’.

## Supplementary Information

**Additional file 1.** RIAT_database_PRGF. Restored baseline data and results.

## Data Availability

All data for which the RIAT authors are directly responsible are included in this published article and its supplementary information files. This RIAT project has used third-party data to generate part of the results presented in the study, which cannot be made openly available. In agreement with the *Trials*’ Availability of data policy, this subject has been explicitly stated across the manuscript and all relevant documents have been provided to *Trials* for purposes of peer review. Readers interested in verifying our analysis are strongly encouraged to approach the corresponding author of the original publication (doi: 10.1016/j.arthro.2012.05.011) in order to obtain the referred underlying data.

## Abbreviations

AE: Adverse event
BMI: Body mass index
CI: Confidence interval
g: Grams
HA: Hyaluronic acid
ITT: Intention-to-treat
NICE: National Institute for Health and Care Excellence
NSAID: Non-steroidal anti-inflammatory drugs
OARSI: Osteoarthritis Research Society International
PP: Per protocol
PRGF: Plasma rich in growth factors
PRP: Platelet-rich plasma
RIAT: Restoring invisible and abandoned trials
SD: Standard deviation
VAS: Visual Analogue Scale
WOMAC: Western Ontario and McMaster Universities Osteoarthritis Index
y: Years
